# Frequency of social burden and underage children in neuro-oncological patients

**DOI:** 10.1007/s00432-023-05338-1

**Published:** 2023-09-07

**Authors:** Marcel A. Kamp, Christiane von Sass, Donjetë Januzi, Maxine Dibué, Katharina Libourius, Anna C. Lawson McLean, Peter Baumgarten, Aaron Lawson McLean, Nazife Dinc, Christian A. Senft

**Affiliations:** Centre of Neuro-Oncology, Department of Neurosurgery, Jena University Hospital, Friedrich-Schiller-University Jena, Jena, Germany

**Keywords:** Malignant brain tumours, Glioma, Glioblastoma, Cerebral metastasis, Children, Underage children, Palliative care, Neuro-palliative care, Psycho-oncological distress

## Abstract

**Objective:**

Brain tumours can cause significant burden for patients and their families, including physical, psychological, and social challenges. This burden can be particularly difficult for patients with malignant brain tumours and those with underage children. However, the frequency of social burden among neuro-oncological patients and the proportion of patients with underaged children is currently unknown. The aim of this retrospective study is to determine the frequency of social and family dysfunction among neuro-oncological patients, the percentage of such patients who have underage children, and to assess their associated burden.

**Methods:**

During a 22-month period, all brain tumour patients were asked to complete a short questionnaire that included epidemiological data, the EORTC-qlq-C30 and -BN20 questionnaire, and the distress thermometer. Data were collected and analysed using Prism 9 for macOS (version 9, GraphPad Prism).

**Results:**

Our analysis included 881 brain tumour patients, of which 540 were female. Median age was 61 years (ranging from 16 to 88 years). Of all patients, 228 suffered from malignant intracranial tumours. More than half of all patients and more than 65% of patients with malignant tumours reported that their illness or medical treatment interfered with their social activities and family life. Almost 30% of patients reported moderate or severe complaints. About 27% of all patients (and 31% of patients with malignancies) expressed moderate or major concerns that their family life could be disrupted. Among the patients with malignancies, 83.5% of patients had a total of 318 children at the time of tumour diagnosis, with a mean age of 33 ± 0.9. Of these patients with malignancies, 38 (17.9%) had a total of 56 underage children at the time of tumour diagnosis, and currently have 53 underage children. Patients with minor children had more financial worries but less interference of their disease with social activities, less psycho-oncological distress, and a more positive outlook into the future (each, *p* < 0.0001). They evaluated their general health status and quality of life in the week prior to their current appointment significantly better (each *p* < 0.0001).

**Conclusion:**

Our study found that 17.9% of patients with malignant brain tumours have underage children. However, having underage children may actually be a positive resource for these patients, as they show lower distress values and better quality of life.

## Introduction

Intracranial tumours that originate in the brain or its surrounding structures represent a significant individual and public health challenge worldwide. They encompass a diverse group of tumour types and are highly heterogeneous. Cerebral metastases are the most common intracranial tumours, with up to 70% of cancer patients who die developing cerebral metastases (Ostrom et al. 2018). Despite significantly improved diagnostic and therapeutic options, brain metastases continue to be associated with high morbidity and mortality rates (Cagney et al. 2017; Sperduto et al. 2010, 2012, 2017). In addition to cerebral metastases, nearly 90,000 new cases of brain and other central nervous system (CNS) tumours were expected to be diagnosed in the USA in 2021 alone (Ostrom et al. 2022). Of these, nearly 70% are classified as benign and approximately 30% as malignant. The second most common brain tumours are (mostly benign) meningiomas, accounting for about 40% of all brain tumours and 55% of non-malignant tumours. Glioblastomas are the most common malignant primary brain tumours, responsible for nearly 50% of malignant primary brain tumours (Ostrom et al. 2022). The median overall survival rate for glioblastoma without any treatment is approximately three months. With multimodal therapy including surgery, radio-/chemotherapy, and early palliative care, prognosis can be improved depending on factors such as age, overall health condition of the patients, tumour location and size, and specific genetic characteristics of the tumour (Mijderwijk et al. 2022; Tamimi and Juweid 2017). Selected subgroups of patients have reported median survival rates of up to 48 years (Herrlinger et al. 2019).

Malignant and benign brain tumours can impose a significant burden on patients, often causing a range of symptoms such as headaches, seizures, and focal neurological deficits. In addition to physical symptoms, brain tumour patients and their loved ones may experience psychological distress with anxiety, depression, and fear of the future (Ley et al. 2022; Rapp et al. 2018; Renovanz et al. 2018; Klein et al. 2001). Receiving a diagnosis of a malignant brain tumour can be a devastating experience for patients and their families as it often leads to a significant decline in the patient’s quality of life and ultimately results in death.

Dame Cicely Saunders’ concept of “total pain” recognizes that complaints are multidimensional, encompassing not only physical pain but also other types of burdens (Ong and Forbes 2005; Clark 1999; Saunders 1964). Along with the physical and psychological components, social and spiritual aspects are integral to managing any serious or life-threatening illness. While physical and psycho-oncological complaints have been widely studied, social and spiritual aspects have received less attention. Moreover, brain tumours can significantly impact patients’ social functioning and family life, especially those with underage children. The effects stemming from tumours, including neurological deficits, cognitive constraints, fatigue, and overall health decline, exert a direct influence on the social engagement and caregiving dynamics within the realm of neuro-oncology patients. Moreover, parents experience a heightened sense of responsibility, especially towards their underage children. Infants, in particular, necessitate targeted focus, guidance, and support in navigating the intricacies of daily existence. For children at the pre-school stage, a harmonious familial environment plays a pivotal role, while school-going children are notably susceptible to their parents’ emotional well-being and adaptive approaches, which subsequently shape their own learning experiences (Shah et al. 2017). We suspected that this special responsibility and knowledge of the prognosis for malignant diseases on the one hand and the obligation along with the prospect of a somewhat unpredictable future for their children on the other, could potentially create a particularly stressful situation for parents with neuro-oncological tumours. Despite the significant burden that these illnesses can impose, little data exist on the prevalence of social and family dysfunction and the proportion of neuro-oncological patients with young dependents. To develop effective supportive measures and adequate treatment plans for these patients and their families, understanding the extent of their burden is crucial.

To address this gap, the present retrospective study aims to determine the frequency of social and family dysfunction among neuro-oncological patients, the percentage of such patients who have underage children, and to assess the associated burden for them.

## Material and methods

### Ethics approval and data availability

We followed the ethical principles outlined in the 1964 Helsinki Declaration and its later amendments, and all procedures involving human participants in this study were approved by the institutional and local ethics committee (study ID: 2022–2809-Daten, ethics committee of the University Hospital, Jena, Germany). Data from this study will be made available upon reasonable request.

### Study design, inclusion and exclusion criteria

For this study, we retrospectively analysed the data of patients who met the following inclusion criteria:

- They received treatment at the tertiary Centre of Neuro-Oncology, Friedrich-Schiller-University, Jena, between 02/2021 and 11/2022.
- They had either neuropathologically or radiologically confirmed brain tumours.
- Patients disclosed whether they had children, the number of children they had, and their children’s ages.
- They completed a distress screening, as well as assessments of palliative care and quality of life.

The exclusion criteria for this study were: (1) Main diagnosis of a spinal tumour or other neuro-oncological tumours than intra-cerebral tumours and (2) incomplete psycho-oncological, palliative care, or quality-of-life screening.

The manuscript has been prepared in accordance with the Strengthening the Reporting of Observational Studies in Epidemiology (STROBE) guidelines, as well as the Equator Network’s recommendations for scientific manuscript preparation (von Elm et al. 2007).

### Standard treatment of brain tumour patients

Treatment for brain tumour patients at our neuro-oncological centre is comprehensive and collaborative. We follow a multimodal, interdisciplinary approach that begins with a detailed consultation and evaluation of the patient’s wishes. Our neuro-oncological tumour board, which includes senior specialists in neurosurgery, neuroradiology, neuropathology, neurology, radiotherapy, and haemato-oncology, provides interdisciplinary advice and recommendations based on the guidelines of the relevant European specialist societies (Goldbrunner et al. 2020; Hoang-Xuan et al. 2023; Le Rhun et al. 2021; Soffietti et al. 2017; Weller et al. 2021).

At our centre, patients with benign brain tumours undergo regular clinical and radiological check-ups using MRI every 4 months. For malignant brain tumours, patients receive their first check-up 6 weeks after surgery or radiotherapy completion, and are subsequently monitored every 3 months using both clinical and radiological assessments. In case of special circumstances, control intervals are shortened or additional examinations such as FET-PET or MR perfusion examinations are carried out.

### Distress, psycho-oncologic, quality of life, and palliative screening

We offered all our inpatients and outpatients to participate in a screening for psycho-oncological issues, palliative medicine needs, and quality-of-life assessment as part of their routine care. An evaluation is conducted at every inpatient and outpatient visit. We use the distress thermometer to screen for psycho-oncological stress and the EORTC-qlq-C30 and -BN20 questionnaires to assess the patient’s quality of life (Rapp et al. 2018; Taphoorn et al. 2010; Schwarz and Hinz 2001). Additionally, we used the NCCN distress thermometer and the core data set of the German Society for Palliative Medicine (DPG) (Bausewein et al. 2005; Stiel et al. 2010; Radbruch et al. 2000a, b; Radbruch et al. 2000a, b).

### Data management and outcome parameters

We retrospectively collected demographic data, such as patients’ diagnoses, age, and gender, as well as information on quality of life, palliative care, and psycho-oncological burden from their charts and questionnaires. For further analysis, quality of life and the global health status was dichotomized into favourable (scores 5–7 on the 7-point Likert scale of the EORTC-qlq-C30 questionnaire) and unfavourable (scores 1–4). The follow-up period lasted until 31 January 2022.

In order to define benign and malignant diseases, we used the World Health Organization (WHO) classification for tumours of the central nervous system (Louis et al. 2021). Based on this definition, we classified grade 3 or 4 tumours according to the WHO classification as being malignant, and WHO grade 1 or 2 tumours as benign (Louis et al. 2021). Cerebral metastases and lymphomas were considered as potentially life-threatening diseases. For some patients with small meningiomas or schwannomas and a watch-and-wait strategy, the diagnosis was made on the basis of neuro-radiological imaging. The criteria of the relevant guidelines and the interdisciplinary assessment in the neuro-oncological tumour board were used as a basis for the classification (Goldbrunner et al. 2016, 2020).

To facilitate further analysis, we divided children into two groups based on their age: minors and adults. We determined the age of majority based on the United Nations (UN) Convention on the Rights of the Child from 1989 and the age limits for majority in most states (United Nations 1989). Specifically, we defined majority as starting from 18 years of age. Upon their initial visit to our neuro-oncological centre, we enquired about the children’s age, and subsequently computed the age at the time of diagnosis.

### Statistical analysis

For continuous data, mean ± standard error of mean was used and for ordinal values, median values and minimum–maximum ranges. Normal distribution of data was assessed using the Shapiro–Wilk test of normality, and were found to be mostly non-normally distributed. To test for a significant difference between two independent groups with non-normally distributed data, the Mann–Whitney *U* test, a nonparametric test, was employed (Wilcoxon 1947, 1946, 1945; Mann and Whitney 1947). For categorical data, median and 25%–75% percentiles were presented. We chose a significance level α of 5% (0.05) for the present study and performed in total ten statistical evaluations (κ = 11). To adjust for multiple comparisons, Šidák’s correction was applied (α_adjusted_ = 1–(1–α)^1/κ^), and a significance level of < 0.0046 was used (Šidák 1967). *P* values between 0.05 and 0.0046 were considered indicative of a tendency towards correlation.

## Results

### Patient cohort

From February 2021 to November 2022, our tertiary neuro-oncology centre treated 881 brain tumour patients who met the inclusion criteria, both as inpatients and outpatients, in a total of 3106 consultations. Of these patients, 540 were female (61.3%) and had a mean age of 61 ± 0.5 years (with a range of 16–88 years). The cohort consisted of 339 patients with meningiomas, 220 with gliomas, and 196 with pituitary tumours, among others (Table 1). A total of 228 patients suffered from malignant or potentially life-threatening intracranial tumours; in this cohort, mean age was 60 ± 1 years (with a range of 16–87 years) and 104 patients (46.6%) were female. The mean follow-up was 48.77 ± 1.09 month (range: 0–476 months).

**Table 1:** Neuro-oncological tumour entities

Diagnosis	WHO-grade	*n*	*n* (total)	*n* (benign)	*n* (malignant)
Cerebral Metastases		64	64		64
Glioma	WHO°1	17	220	17	
	WHO°2	61		61	
	WHO°3	40			40
	WHO°4	102			102
Meningioma	WHO°3	8	339		8
	WHO°2	38		38	
	WHO°1	293		293	
Other	Other	21	21	16	5
Pituitary gland adenoma	WHO°1	196	196	196	
PCNSL		9	9		9
Schwannoma	WHO°1	32	32	32	
		881		653	228

The table provides an overview of the tumour types and entities diagnosed in the patient cohort analysed here

### Distress and future expectancy

The median distress score was 5 out of 10 for both the entire cohort (25th–75th percentile range 2–7) and for patients with malignancies (25th–75th percentile range 3–7) at the time of initial presentation in our tumour centre. More than three quarters of all patients reported feeling uncertain about their future, and nearly 60% of them reported a worsened outlook on the future (for exact numbers, see Table 2). Among patients with malignancies, 85% felt uncertain about their future, and 50% reported a worsened outlook on the future. The overall health condition and quality of life were rated as unfavourable in 23% and 24% of all patients and 27% and 40% of patients with malignant tumours, respectively.

**Table 2:** Disruption of family life, social burden, and quality of life

	Has your physical condition or medical treatment interfered with your family life?		Has your physical condition or medical treatment interfered with your social activities?		Has your physical condition or medical treatment caused you financial difficulties?		Did you feel uncertain about the future?		Did you feel you had setbacks in your condition?		Were you concerned about disruption of family life?		Did your outlook on the future worsen?			How would you rate your overall health during the past week?		How would you rate your overall quality of life during the past week?	
Questionnaire	EORTC-qlq-C30		EORTC-qlq-C30		EORTC-qlq-C30		EORTC-qlq-BN20		EORTC-qlq-BN20		EORTC-qlq-BN20		EORTC-qlq-BN20			EORTC-qlq-C30		EORTC-qlq-C30	
	n	%	n	%	n	%	n	%	n	%	n	%	n	%		n	%	n	%
Entire Cohort																			
Complaints															Complaints				
Not at all	832	42.7%	633	45.3%	956	68.6%	428	22.3%	694	36.6%	869	45.9%	774	40.9%	7—excellent	104	5.3%	131	0.07
Few / slight complaints	567	29.1%	382	27.4%	242	17.4%	673	35.1%	629	33.2%	524	27.7%	613	32.4%		399	20.4%	442	0.23
																546	27.9%	493	0.25
																460	23.5%	432	0.22
Moderate complaints	327	16.8%	227	16.3%	134	9.6%	501	26.1%	361	19.0%	314	16.6%	352	18.6%		293	15.0%	284	0.14
Severe complaints / major impairment	222	11.4%	154	11.0%	61	4.4%	318	16.6%	213	11.2%	187	9.9%	153	8.1%		107	5.5%	128	0.07
															1—very bad	48	2.5%	52	0.03
Complete and unambiguous information available in consultations	1948		1396		1393		1920		1897		1894		1892		Complete and unambiguous information available in consultations	1957		1962	
Patients with malignant tumours																			
Complaints															Complaints				
Not at all	200	29.0%	139	29.9%	269	58.4%	98	14.6%	192	28.7%	218	32.9%	219	25.0%		24	3.5%	30	3.7%
Few / slight complaints	213	30.9%	148	31.8%	103	22.3%	196	29.2%	229	34.2%	215	32.5%	228	26.0%		115	16.7%	132	16.1%
																203	29.5%	169	20.7%
																160	23.3%	156	19.1%
Moderate complaints	156	22.6%	99	21.3%	69	15.0%	217	32.3%	141	21.0%	128	19.3%	150	17.1%		117	17.0%	169	20.7%
Severe complaints / major impairment	120	17.4%	79	17.0%	20	4.3%	160	23.8%	108	16.1%	101	15.3%	66	7.5%		46	6.7%	132	16.1%
															1—very bad	23	3.3%	30	3.7%
Complete and unambiguous information available in consultations	689		465		461		671		670		662		663		Complete and unambiguous information available in consultations	688		818	

The study examined the impact of family, social life, and overall quality of life on neuro-oncological patients using various questionnaires including EORTC-qlq-c30, -BN30, MIDOS, and modified distress thermometer. The results of the screening are presented in Table 2

### Family life and social burden

Out of the total of 675 patients with comprehensive data, 138 individuals (20.4%) disclosed that they resided alone without any family members or companions and 48 out of 251 patients with malignant tumours lived alone. Additionally, 617 patients (which accounts for 80% of all patients, with information available for 773 patients) were in committed relationships, including 229 out of 280 patients with malignancies (81.7%) who had a complete set of information.

Based on responses to the EORTC-qlq-C30 and -BN20 questionnaires, over half of all patients and more than 65% of those with life-threatening tumours indicated that their illness or treatment disrupted their social activities and family life upon first presentation at our tumour centre (for exact numbers, see Table 2). Almost 30% of patients had moderate or severe complaints, as detailed in Table 2. About 27% of all patients (and 31% of patients with malignancies) expressed moderate or major concern that their family life could be disrupted. Approximately 15% of patients (17% of patients with malignancies) faced moderate or major financial difficulties due to their illness.

### Children

**Fig. 1:**
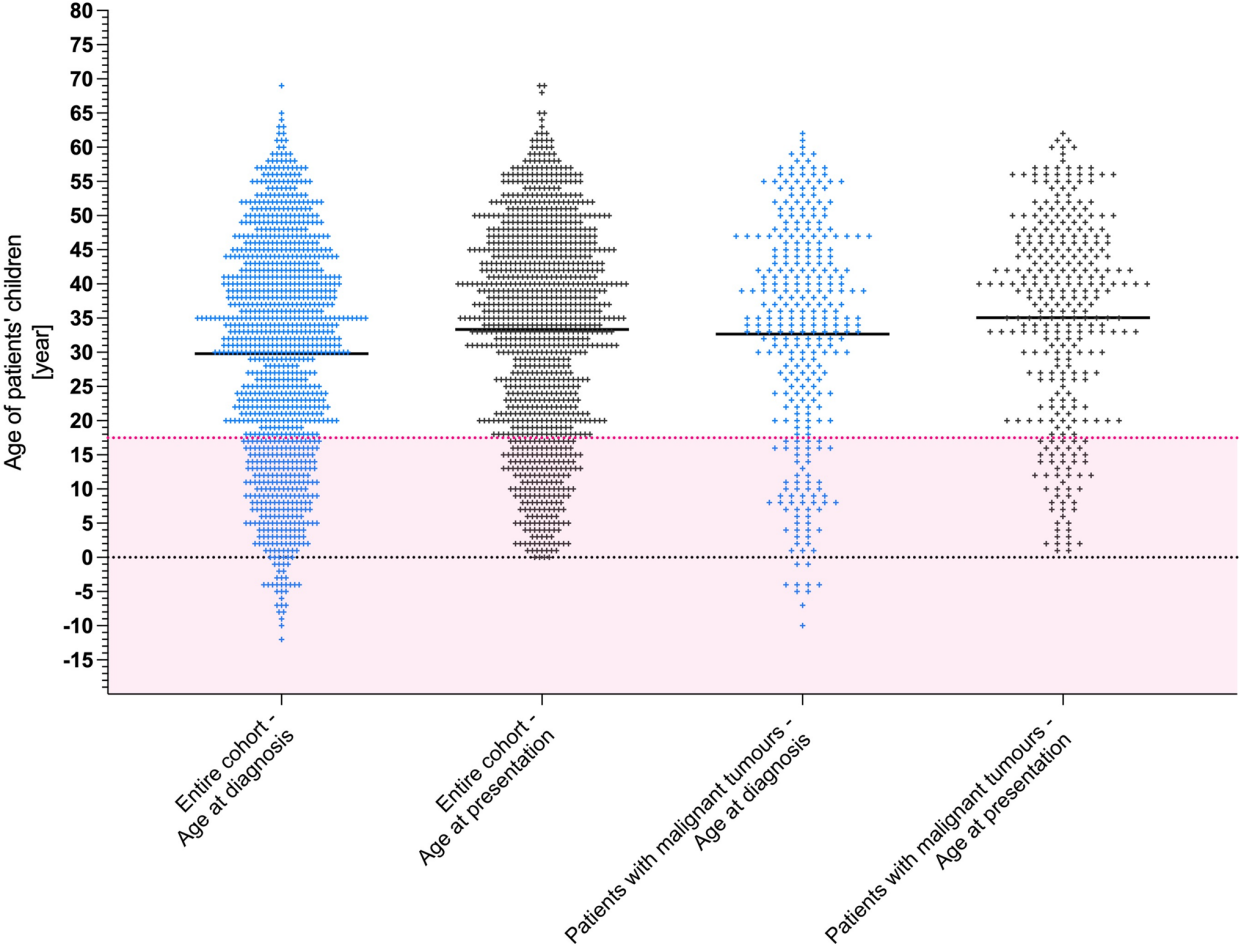
Age distribution of neuro-oncological patients. The figure illustrates the age distribution of the children of neuro-oncological patients, displaying data for the entire cohort and patients with malignant tumours as well as at the time of tumour diagnosis and their first presentation in our department. It is important to note that some children were born after the patient’s diagnosis, which should be reflected in the partly negative age values in the diagrams indicating their age at the time of diagnosis

Out of 881 patients, 61 (6.9%) declined to provide information about their children, as well as 16 (7%) out of 228 patients with malignant diseases. A total of 162 patients of the entire cohort (19.6%) reported having no children. On average, each patient had 1.5 ± 0.04 children, with a range of 0–7 children. At the time of diagnosis, the total cohort had a total of 1083 children. At the time of their presentation to our clinic, patients’ children had a mean age of 33 ± 0.5 years, while at the time of diagnosis of intracranial neuro-oncological disease, their children’s mean age was 30 ± 0.5 years (Fig. 1). At the time of the parent’s tumour diagnosis, 231 patients (28.2%) had underage children.

Among the patients with malignancies, 212 provided complete information about their children. Of these, 177 patients (83.5%) had a total of 318 children at the time of tumour diagnosis, with a mean age of 33 ± 0.9 years (35 ± 0.8 years at the time of presentation to our clinic). Thirty-eight patients with malignancies (17.9%) had a total of 56 underage children at the time of tumour diagnosis, and currently have 53 underage children.

### Burden of parents with malignant diseases and minors

**Fig. 2:**
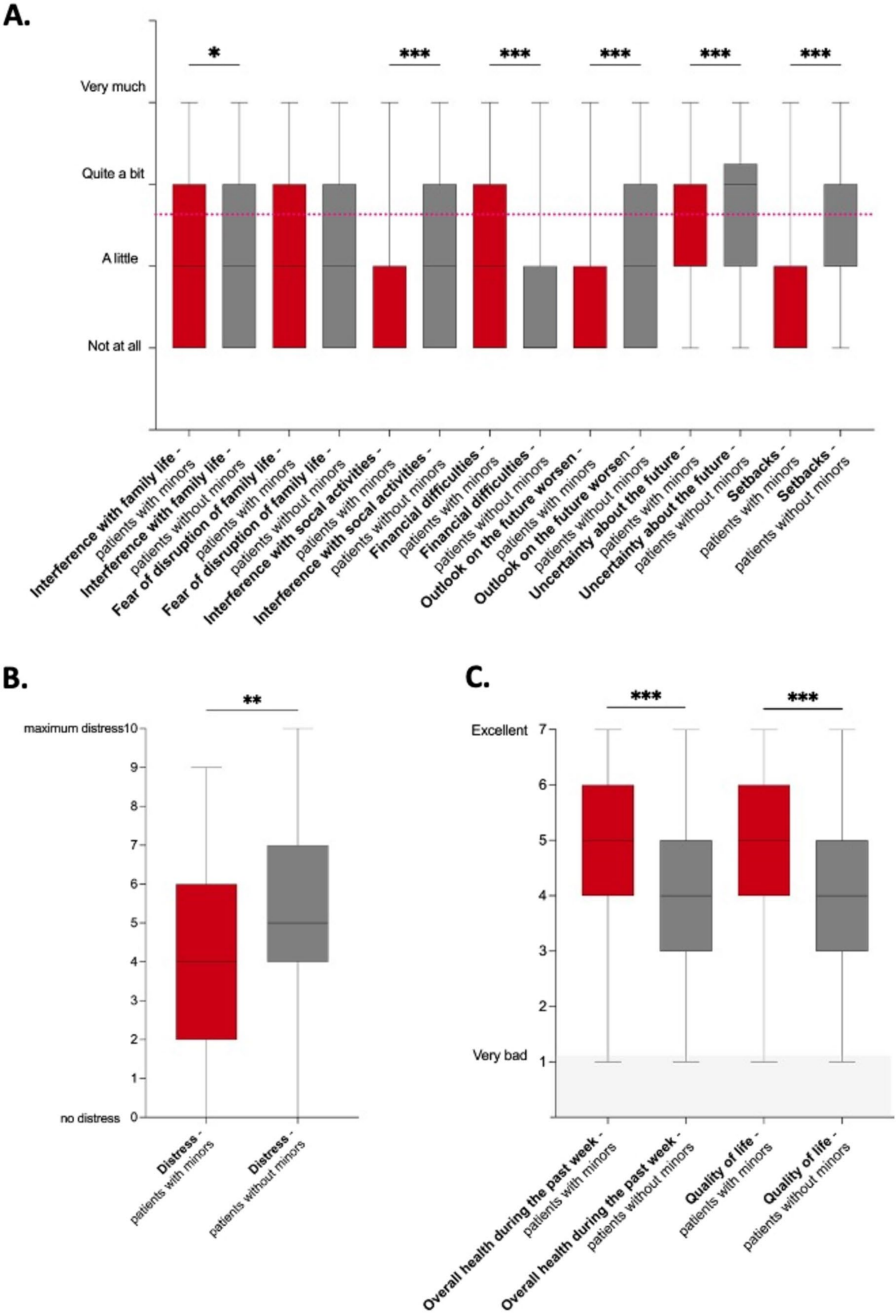
Social burden, family life, overall quality of life, and distress of neuro-oncological cancer patients with and without dependent children. The figure summarizes the results of an evaluation of the social life, family dynamics (**A**), distress levels (**B**), and overall quality of life/global health status (**C**) of neuro-oncological patients with malignant tumours, both with and without dependent children

This study aimed to investigate whether underage children with a malignant disease experience a particular psycho-oncological burden. To achieve this, we compared the psycho-oncological burden of patients with malignant disease and underage children to that of patients with malignant disease but no underage children at the time of presentation in our tumour centre (Fig. 2). We found that patients with minor children had more financial worries (median 2 vs. 1, range 1–4; *p* < 0.0001) but less interference of their disease with social activities (median 2 for both groups, *p* < 0.0001), less psycho-oncological distress (median 4 vs. 5, range 1–10, *p* < 0.003), fewer setbacks (median 2 for both groups, range 1–4, *p* < 0.0001), and better future prospects (median 1 vs. 2, range 1–4, *p* < 0.0001). They were less limited in doing either work or other daily activities (median 2 for both groups, range 1–4, *p* < 0.0001) and in pursuing hobbies or other leisure time activities (median 2 for both groups, range 1–4, *p* < 0.0001). Their general health status and quality of life (median 5 vs. 4, range 1–7; *p* < 0.0001 for both parameters) were significantly better in week prior to the appointment. Patients with minor children also reported a better family life (median 2 for both groups, *p* < 0.003), but they had the same level of fear of disruption of family life as patients without minors (median 2 for both groups, *p* < 0.5).

## Discussion

The main results of our present study are: (1) More than half of all neuro-oncological patients reported to suffer from a moderate or severe interference of their illness with their social activities and family life. (2) 17.9% of patients with malignancies of our cohort had minors and averaged 1.5 underage children at the time of tumour diagnosis. (3) Patients with minors and malignant neuro-oncological tumours have less interference of their disease with social activities, less psycho-oncological distress, better future prospects, a better general health status, and quality of life in the present cohort.

Research on the frequency of underage children of cancer patients and the psycho-oncological and social burden that parents with neuro-oncological cancer face is comparatively limited. Our study is the first to evaluate the prevalence of minors among patients with malignant neuro-oncological tumours, which we found to be 17.9%. These results are consistent with previous findings that indicate between 14% and 24.7% of cancer patients have minor children, depending on the age range of the sample (Inhestern et al. 2021). However, these studies did not specifically focus on malignant neuro-oncological tumours, but rather cancer in general.

We hypothesized that parents of minors with cancer would face a heavy burden in terms of their psycho-oncological and social well-being. A systematic review of the impact of cancer on the mental health of parents with teenage children included a total of 54 articles from 36 different studies. The review found that between 7 and 83% of patients met criteria for probable clinical depression, while 19%–88% met criteria for probable anxiety disorders (Johannsen et al. 2022). The authors concluded that the disease and its consequences have a significant impact on the mental health of parents who are suffering from cancer with children (Johannsen et al. 2022). These findings are supported by a previous study not included in the review: a multi-institutional, prospective cohort study with 668 patients with advanced cancer who were parenting with dependent children and those who were not. Patients with minors were found to be significantly more worried, more likely to meet the criteria for panic disorder diagnosis, and less peaceful (Nilsson et al. 2009). However, it should be noted that these studies can only be compared with our present analysis to a limited extent. Specifically, they included cancer patients in general, focused more on psycho-oncological rather than social parameters, and some studies did not compare cancer patients with and without children (Johannsen et al. 2022).

Our study also showed that patients had a disease-related limitation of quality of life, social life, and family life. Approximately 25% of all patients and cancer patients reported an unfavourable overall health condition in the past week. Twenty-four per cent of the entire cohort and 40% of the patients with malignant tumours rated their quality of life in the week prior to their appointment as “poor”. In addition, more than half of the patients and 65% of those with cancer experienced moderate or severe interference of their illness with their social activities and family life. The majority (85%) of patients with malignancies expressed uncertainty about their future, and half (50%) reported a worsened outlook for the future. However, our findings differ from the aforementioned results. Our patients suffering from malignant neuro-oncological tumours who have underage children reported significantly less interference of their disease with social activities, less psycho-oncological distress, better future prospects, a better general health status, and quality of life when compared to cancer patients without dependent children. In our study, minor children or having a family life with minor children appeared to be a resource rather than a burden. This result is also consistent with previous results: Ernst and colleagues assessed how cancer patients’ quality of life changed over time, using a questionnaire during treatment (T1) and two years later (T2) (Ernst et al. 2012). The study compared two groups: patients with children under 18 years (*n* = 41) and those without children (*n* = 28). Both groups reported low quality of life at T1, but at T2, the group with children reported better quality of life on most dimensions. However, the authors discussed that being female and having a partner might have a greater impact on quality of life than being a parent (Ernst et al. 2012). In a previous study conducted in the Netherlands, it was shown that cancer patients with dependent children aged between 4 and 18 years experienced reduced but improved psychosocial functioning over time, and reported less stress compared to the general population (Gazendam-Donofrio et al. 2008). However, a comparison group of cancer patients without dependent children was not included in this study. We are unable to fully explain the differences in study results regarding whether children are a burden or a resource for parents with cancer. Several explanations are conceivable. Firstly, different types of cancer can have varying effects on the impairment of quality of life and social and family functioning, and most studies have included cancers in general (Gazendam-Donofrio et al. 2008). Our study, on the other hand, specifically focused on neuro-oncological diseases, and we have observed that radiotherapy and oral chemotherapy, which are often used in malignant neuro-oncological diseases, are relatively well tolerated, particularly in the initial stages (Berger et al. 2021; Rapp et al. 2018). Secondly, our study focused more on social and family impairments, while other studies have concentrated more on psycho-oncological distress. Thirdly, the study by Ernst and co-workers and our study were conducted in the same region, and regional, cultural factors and family ties may play an important role in quality of life, family, and social life. Lastly, both studies used the same instrument, namely the EORTC-qlq-C30 questionnaire (and in our study, we also used the EORTC-qlq-BN20 questionnaire) to measure quality of life, social, and family impairments. However, other studies have often employed different instruments, such as the SF-8.

Modern treatment for neuro-oncological patients goes beyond anti-tumour therapy and should address all aspects of the total pain concept, including physical, psychological, social, and spiritual complaints. Palliative medicine is no longer solely viewed as end-of-life care, but rather as an early intervention to complement actual tumour therapy. The goal of therapy is to prevent and relief “suffering through the early identification, correct assessment and treatment of pain and other problems, whether physical, psychosocial or spiritual” (World Health Organization 2002). Social and family life burden are important factors to consider in preventing and treating suffering. Social issues encompass various aspects of care and engagement in both social and professional spheres. A recent study revealed that approximately 70% of individuals diagnosed with WHO°2 and °3 glioma, following surgery and adjuvant treatment, were able to reintegrate into the workforce. The median duration until resuming work was 8 months (Senft et al. 2020). In addition to multidimensional treatment including social issues and implementation of patient-centred outcome parameters, it is essential to understand the prevalence of neuro-oncological patients with young dependents impacted by these illnesses to develop supportive measures that can assist patients and their families in managing the many challenges they may face. Further studies and new treatment approaches should address social and family life dysfunction of neuro-oncological patients.

### Limitations

We acknowledge several limitations of our present analysis. Firstly, all the data were derived from a retrospective, single centre study. Secondly, sample size calculation was lacking, but as a retrospective cohort was identified, post-hoc sample size calculation would be unusual. Future prospective studies should include a sample size calculation to draw more confirmative conclusions. Since the aim of the analysis was to determine the frequency of social and family stress on neuro-oncological patients, we have not performed a multivariate analysis, created a clinical prediction model for quality of life, nor have we assessed the burden carried by underage children. As our aim was to evaluate the impact of brain tumours on social functioning and distress, we used frequencies of each single item of the EORTC-qlq-C30 questionnaire and not the sum scores or subscore for further analysis (Aaronson et al. 1993). It should also be noted that we did not gather data on additional living conditions, e.g. single-parent households or individuals living alone without children. Additionally, the age of children might correlate with cancer patient's burden, e.g. that patients with younger children might suffer from higher distress; we have not accessed the impact of children's age on the distress of neuro-oncological parenting patients. These unexamined parameters could potentially influence the results as well. The potential younger age of patients with underage children might also influence the results, e.g. difference of age as reason for all the differences reported. Moreover, the present analysis compromised a very heterogeneous patient collective. The estimation of burden of parents with malignant diseases and minors based on 38 patients out of an entire cohort of 881 brain tumour patients and 228 with malignancies. However, it was the goal of our research to also determine the frequency of underaged children and social impairment caused by brain tumours. We did not analyse if and how different diagnoses and treatment protocols may affect the assessed parameters such as quality of life, social activities, or family life. Likely, the different disease stages, such as following first-line treatment, during follow-up, or end-of-life care, may differently affect psycho-oncological and social burden. The data arose from a tertiary neuro-oncological centre in Central/Eastern Germany. The geographical area from which we drew our patients is partially rural, resulting in considerable distances between patients' homes and the neuro-oncological center. The region was part of the former German Democratic Republic and a former communist country with a sometimes-lower socio-economic status than other regions of Europe. Thus, findings in this population cohort are not necessarily transferable to other regions in Europe and worldwide. Again, regional, cultural factors, and family ties may play an important role in quality of life, family, and social life. Fifthly, in this study, we pooled the quality of life at different points in the course of the disease, as opposed to tracking quality of life, social and family life over time in individuals, as done in other studies (Ernst et al. 2012). Certainly, the timing point of the illness will have an impact on social and family life, as well as quality of life. Sixth, we examined the social and family burden of cancer patients, particularly those with minor children. We did not differentiate whether the parent was the mother or the father. However, affected mothers and fathers may have different levels of burden. Finally, while our study focused on evaluating the social life, family functioning, and quality of life of neuro-oncological patients with and without dependent children, we did not assess the distress experienced by the children themselves. To gain a more comprehensive understanding of the impact of neuro-oncological diseases on families, future studies should examine the burden of the disease on both the patients and their children. Such studies could shed light on the unique challenges faced by families affected by neuro-oncological diseases and help inform the development of more effective support and care strategies.

## Conclusion

Many neuro-oncological patients experience moderate to severe interference in their social activities and family life. In our cohort, 17.9% of malignant brain tumour patients had minors. However, patients who suffer from malignant neuro-oncological tumours and have minors appear to experience less interference with their social activities, lower psycho-oncological distress, better future prospects, and better overall health status and quality of life. Future study endeavours should further explore the extent to which children can serve as a source of support for neuro-oncological patients. Furthermore, these studies might identify further potential confounders and assess innovative strategies aimed at enhancing the social engagement and familial well-being of individuals with neuro-oncological tumours.”
